# Investigation on antigen-specific T-cell responses induced by outer membrane vesicles from *Escherichia coli Δ60* strain

**DOI:** 10.3389/fimmu.2025.1633961

**Published:** 2025-10-14

**Authors:** Michele Tomasi, Lorenzo Croia, Ilaria Zanella, Assunta Gagliardi, Giulia Boscato Sopetto, Mattia Benedet, Gabriele Di Lascio, Gaia Gambini, Alvise Berti, Riccardo Corbellari, Guido Grandi, Alberto Grandi

**Affiliations:** ^1^ Toscana Life Sciences Foundation, Siena, Italy; ^2^ Department of Cellular, Computational and Integrative Biology (CIBIO), University of Trento, Trento, Italy; ^3^ BiOMViS srl, Siena, Italy

**Keywords:** outer membrane vesicles (OMVs), vaccines, CD4 T cell, CD8 T cell, cell mediated immunity

## Abstract

There is a growing interest in the exploitation of bacterial outer membrane vesicles (OMVs) for the design of vaccines and novel antitumor immunotherapeutic products. Such interest is motivated by their potent immunostimulatory properties, which promote elevated immune responses against heterologous antigens combined with OMVs by genetic engineering, chemical coupling, or absorption. However, for a full exploitation of OMVs, a few questions remain to be fully addressed: what is the appropriate ratio of OMVs/heterologous antigen needed to obtain an optimal antigen-specific immune response? To what extent do OMV endogenous proteins interfere with or favor antigen-specific immunity? Using OMVs derived from our *Escherichia coli* Δ*60* (*E*. *coli Δ60*) strain, we recently addressed these questions, focusing on the humoral immune responses, and we determined the concentrations of the OMV-associated proteins necessary and sufficient to elicit saturating levels of specific antibodies. In this work, we focused on cell-mediated immunity. We show that, because of the numerous OMV-associated MHC II epitopes, OMV immunization elicited detectable levels of IFN-γ^+^ epitope-specific CD4^+^ T cells provided that epitope concentrations were >10% of the total OMV proteins (w/w). Such elevated concentrations could be achieved by mixing synthetic peptides with OMVs but not by genetic manipulation of OMVs. By contrast, most likely thanks to the cross- help of the polyclonal CD4^+^ T cell population, elevated frequencies of epitope-specific CD8^+^ T cells were found even when MHC I epitopes were present at concentrations lower than 1% of the total OMV proteins. Our data provide a mechanistic insight of the OMV-mediated immune responses and have important implication in vaccine design.

## Introduction

Outer membrane vesicles (OMVs) are non-replicating spherical particles ranging from 20 to 250 nm in diameter, released by all Gram-negative bacteria through the budding out of the external membrane (1). Consistent with their origin, OMVs primarily consist of lipopolysaccharides (LPS), glycerophospholipids, outer membrane and periplasmic proteins, and peptidoglycan. Moreover, and somehow difficult to explain, the presence of nucleic acids has also been reported (2, 3). OMVs exert several fascinating biological functions such as biofilm formation, inter- and intraspecies communication, toxin delivery, and enzyme and gene exchange with other bacterial species (1–5).

OMVs are gaining attention as a promising vaccine platform against infectious diseases and cancer due to several favorable properties. First, they carry numerous microbe-associated molecular patterns (MAMPs) such as LPS, lipoproteins, and peptidoglycan, which altogether and in a concerted manner elicit a strong Th1 type of immune response (6–11). Second, using molecular and synthetic biology techniques, the OMV protein content can be manipulated, allowing the accumulation of heterologous antigens and epitopes in the lumen or on the surface of OMVs (12–23). As a result, due to their intrinsic adjuvanticity, immunization with engineered OMVs elicits potent B- and T-cell responses against foreign components (6, 23–28). Third, using either detergent extraction or OMV-overproducing strains, OMV production can be efficiently scaled up from laboratory to industrial levels with yields higher than 100 mg/L of culture (29).

OMV-based vaccines can be broadly categorized into two categories: vaccines formulated with OMVs purified from a Gram-negative bacterial pathogen (“homologous OMV vaccines”) and vaccines formulated with OMVs derived from a Gram-negative bacterium properly engineered to accumulate foreign antigens in the vesicular compartment (“heterologous OMV vaccines”). Vaccines belonging to the first category are already available for human use (12). They are very effective but have an intrinsic limitation that can be developed only against Gram-negative bacterial pathogens. In contrast, vaccines belonging to the second category hold the potential to target a wide range of pathogens, including Gram-positive bacteria and viruses, and recent data also show their possible application in cancer immunotherapy (14, 19, 23, 25, 27, 28, 30–32).

Heterologous OMV vaccines have not reached the clinics yet. Not only they need some technical optimization but also some aspects related to their mechanisms of action remain to be fully understood. For instance, one open question is how and to what extent the efficacy and safety of heterologous OMV vaccines can be affected by the OMV endogenous proteins. OMVs carry a conspicuous number of endogenous proteins. Depending on the bacterial species, growth conditions, quality of the OMV preparation, and analytical methods used for protein identification, protein contents ranging from 100 to 400 unique protein species have been reported (33). In the context of vaccination, these proteins might negatively or positively influence the immune responses against the heterologous vaccine antigen(s). On the one hand, being immunogenic themselves, they could potentially induce unwanted reactions and could “dilute” the immune responses toward the target antigen(s), thus reducing the potency of the vaccine. On the other hand, they could enhance the immunogenicity of heterologous antigens, for instance, by providing cross T cell help.

In an attempt to fill this knowledge gap, we recently investigated the antibody responses elicited by the proteins present in the OMVs of *E. coli Δ60* (OMVs_Δ60_), a strain created in our laboratory having a unique property to release abundant quantities of vesicles deprived of a conspicuous number of endogenous proteins (34). Upon immunization, we found that most of the proteins present at concentrations ≥1% of total proteins (w/w) induce protein-specific antibody responses, regardless of their localization within the vesicles (35). The presence of aluminum salts usually, but not necessarily always, improves the total protein-specific IgG titers. Moreover, those proteins expressed at concentrations higher than 4% of the total OMV proteins elicit “saturating” IgG titers even when mice are immunized with an amount of OMVs as low as 1 µg/dose. In mice, increasing the quantity of administered OMVs has minimal impact on the total protein-specific IgG titers. Overall, these results have important implications for vaccine design and for setting the vaccine doses. When a heterologous antigen accumulates in the vesicles at concentrations >5% of the total OMV proteins, with expression levels that are routinely reached using technologies currently available (22), a few micrograms of vesicles are expected to be sufficient to guarantee vaccine effectiveness.

Regarding OMV-induced cell-mediated immunity, studies investigating the specificity of CD4^+^ and CD8^+^ T-cell responses elicited by OMV-associated MHC I and MHC II epitopes are still limited. In particular, the profile of the T-cell population induced by OMV immunization and the epitope concentration needed to allow epitope recognition by naive T cells remain unclear. Zhang and coworkers used the *Salmonella* autotransporter protein MisL to display a specific MHC class II chicken ovalbumin peptide (OVA-CD4) on the surface of *Salmonella* OMVs. They showed that the engineered OMVs induced OVA-specific T-cell responses; however, no quantitative analysis was reported (36). In two independent works, C57BL/6 mice challenged with B16 melanoma cells expressing chicken ovalbumin (OVA) were vaccinated with *E. coli* OMVs engineered with different epitopes including the MHC I OVA_257–264_ epitope. Tumor growth was substantially inhibited in vaccinated mice, but no quantitative analysis aimed at correlating the concentration of the epitopes in OMV quantities and OVA-specific T-cell frequencies was described (19, 37). In a different study, OVA-specific CD8^+^ T cells with cytotoxic activity were induced by subcutaneous and intramuscular immunization of mice with a mixture of 50 µg of *Neisseria meningitidis* OMVs and 1 mg of whole ovalbumin protein. The administration route did not influence the magnitude of the OVA-specific CD8^+^ T-cell response (38), but again, no quantitative data were provided. Finally, we recently decorated *E. coli* OMVs with different T-cell epitopes (25, 27, 28, 32), and we showed that 20 µg of OMVs were sufficient to elicit T-cell responses and to inhibit tumor growth in different mouse models. Again, the correlation between epitope-specific T-cell frequencies and the levels of OMV/epitope expression was not investigated.

The goal of this work was to investigate how different levels of MHC I and MHC II epitopes present in OMVs_Δ60_ affect the epitope-specific T-cell responses in mice. In particular, we sought to establish the minimum amount of epitope necessary to induce detectable levels of epitope-specific T cells using ELISpot and flow cytometry analyses.

## Results

### Epitope-specific CD4^+^ T cells induced by OMVs_Δ60_

The bioinformatic analysis of the OMV proteins predicts the high abundance of MHC II epitopes. Therefore, assuming that most of the OMV proteins enter the MHC II presentation pathway, OMV immunization is expected to induce a highly polyclonal T-cell population, with the frequency of each T-cell clone being relatively low. As a consequence, sufficiently high frequencies of a T-cell clone specific for a particular epitope can be induced by OMV immunization provided that the concentration of such epitope is high enough to successfully compete with other epitopes. The concentrations necessary and sufficient to induce epitope-specific T-cell clones need to be thoroughly investigated.

**Figure 1:**
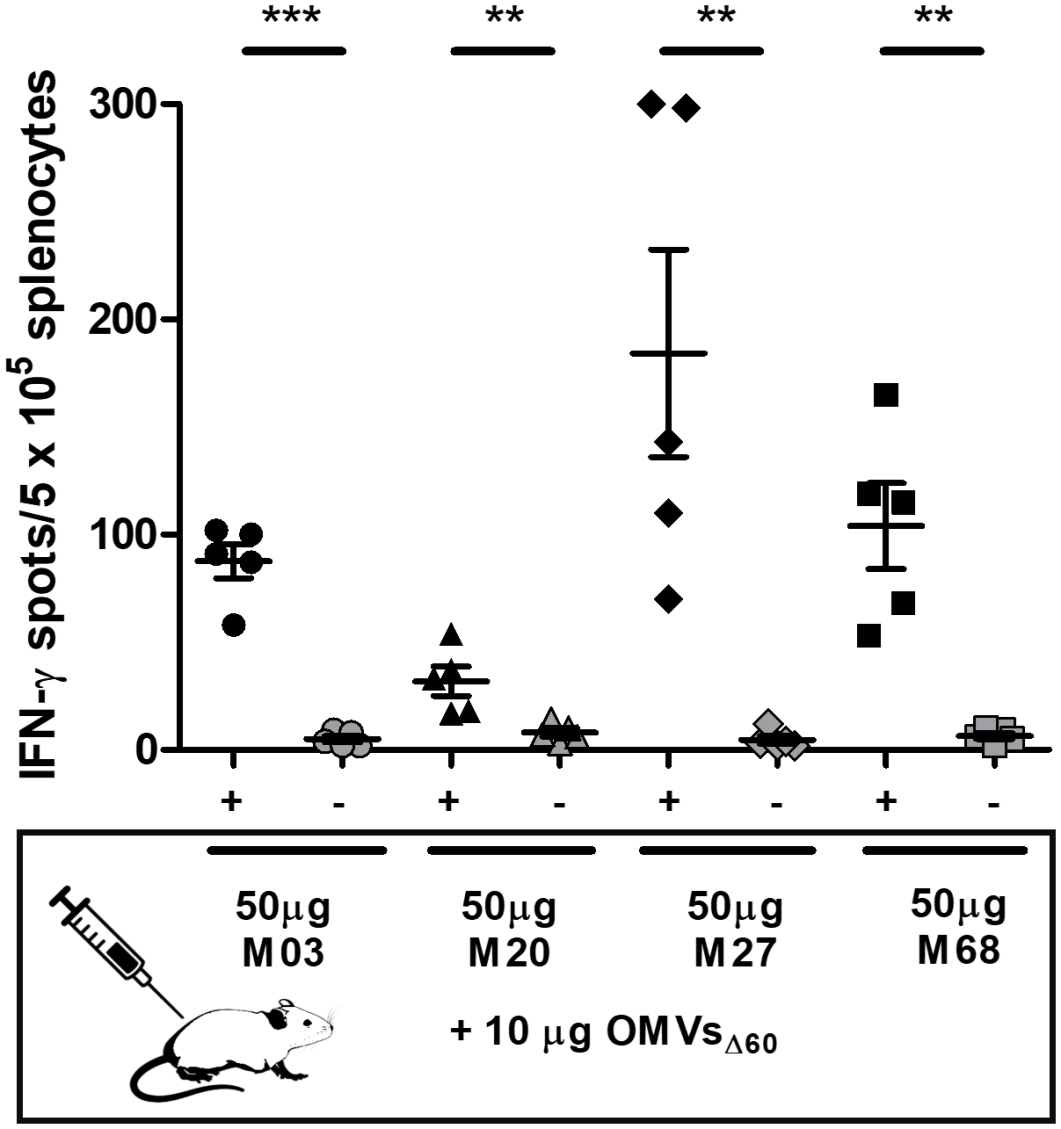
Frequencies of epitope-specific INF-γ^+^ CD4^+^ T cells induced in mice by immunization with OMVs_Δ60_ decorated with different MHC II epitopes. BALB/c mice were immunized with four different formulations of OMVs_Δ60_ (10 µg) mixed with different synthetic peptides (50 µg), each carrying one of the MHC II-restricted epitopes: M03, M20, M27, and M68. Mice were immunized intraperitoneally (i.p.) on day 0 and day 7, and the splenocytes, harvested on day 12, were stimulated with either 5 µg/mL of the corresponding peptides used for the immunization (black) or 5 µg/mL of an unrelated peptide as a control (gray). Statistical significance was calculated using an unpaired, two-tailed Student’s *t*-test. **P < 0.01; ***P < 0.001.

To address this question, we first selected four MHC II epitopes—M03, M20, M27, and M68—experimentally demonstrated to be immunogenic in BALB/c mice. These epitopes were first described by Kreiter and coworkers and were identified by bioinformatics analysis of the genome of the CT26 tumor cell line. The authors showed that a synthetic RNA encoding such epitopes elicited high frequencies of epitope-specific CD4^+^ T cells in BALB/c mice and that these T cells were capable of protecting mice from being challenged with the CT26 cell line (39). With this information, we chemically synthesized the 27-amino-acid-long peptides encompassing the M03, M20, M27, and M68 epitopes, and BALB/c mice were immunized i.p. with 10 µg of OMVs_Δ60_ mixed with 50 µg of one of the four synthetic peptides (in 200 µL of PBS). Each mixture was injected twice, 1 week apart, and 5 days after the second immunization, the frequencies of IFN-γ^+^ T cells in the spleens of each immunized animal were analyzed by the ELISpot assay. As shown in **Figure 1**, all formulations elicited epitope-specific T cells, with frequencies ranging from 50 to 200 IFN-γ^+^ T cells/5 × 10^5^ splenocytes. The observed frequencies confirmed our previous data using flow cytometry analysis of splenic IFN-γ^+^ CD4^+^ T cells from mice immunized with OMVs purified from a different *E. coli* strain (32) and appeared to be in line with the published CD4^+^ T-cell frequencies using synthetic mRNA or synthetic peptides + Hiltonol® (39).

**Figure 2:**
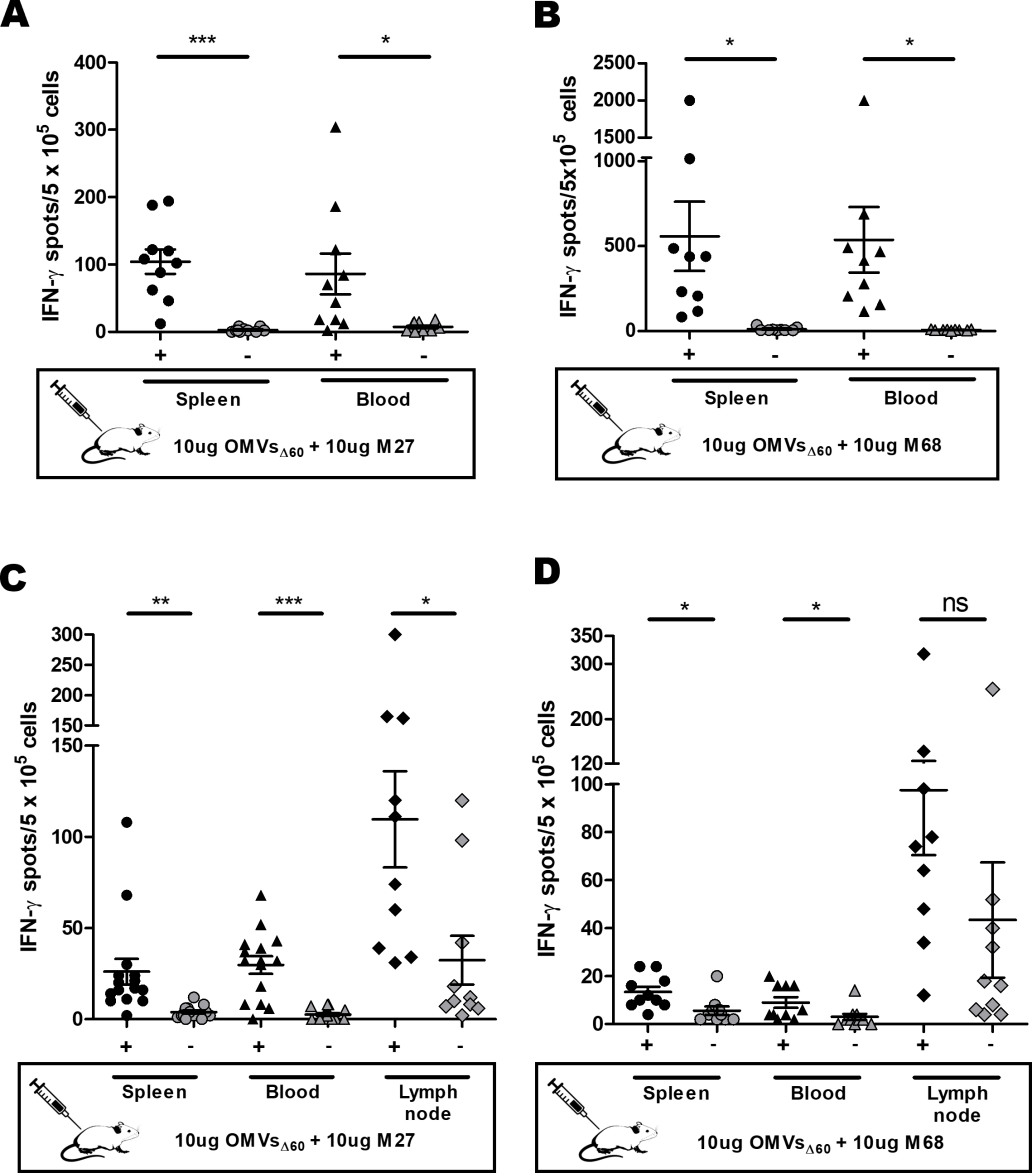
Analysis of M27- and M68-specific CD4^+^ T-cell frequencies induced through i.p. and s.c. immunization. **(A, B)** Frequencies of peptide-specific IFN-γ^+^ CD4^+^ T cells induced by a three-dose i.p. vaccination with OMVs_Δ60_ + M27 **(A)** or M68 **(B)** peptides. Mice were i.p. immunized three times, 1 week apart, with 10 µg of OMVs_Δ60_ adsorbed with 10 µg of M27 or M68 peptides. Splenocytes and PBMCs harvested 5 days after the last vaccine injection were stimulated *in vitro* with either 5 µg/mL of the vaccination peptide (M27 or M68) or 5 µg/mL of an unrelated peptide as a control (gray). **(C, D)** Frequencies of peptide-specific IFN-γ^+^ CD4^+^ T cells induced by a three-dose s.c. vaccination with OMVs_Δ60_ + peptide. Mice were immunized s.c. with 10 µg of OMVs_Δ60_ adsorbed with 10 µg of M27 **(C)** or M68 **(D)** peptides. Splenocytes, PBMCs, and lymph nodes were collected 5 days after the last injection and restimulated with either 5 µg/mL of the vaccination peptide (M27 or M68, black) or 5 µg/mL of an unrelated peptide as a control (gray). Statistical significance was calculated using an unpaired, two-tailed Student’s *t*-test. ns, not significant; *P < 0.1; **P < 0.01; ***P < 0.001.

Next, M27 and M68, the most immunogenic epitopes, were used to carry out two additional sets of experiments. First, we measured T-cell frequencies in both the spleen (the main draining secondary lymphoid organ of the peritoneum, together with the mediastinal and tracheobronchial lymph nodes) and blood, using a three-immunization regimen. As shown in **Figures 2A, B**, M27- and M68-specific T cells were detected in both compartments, and in the case of M68-specific T cells, the three-immunization regimen appeared to improve the frequencies of the splenic IFN-γ^+^ CD4^+^ T cells. In the second set of experiments, the animals were subcutaneously immunized three times at a 1-week interval (**Figures 2C, D**), and the IFN-γ^+^ T cells were measured in the popliteal lymph nodes, spleen, and blood 5 days after the third immunization. Frequencies of approximately 100 specific IFN-γ^+^ T cells/5 × 10^5^ cells were measured in the lymph nodes of mice immunized with both peptides. Epitope-specific T cells were also found in the blood and spleen.

**Figure 3:**
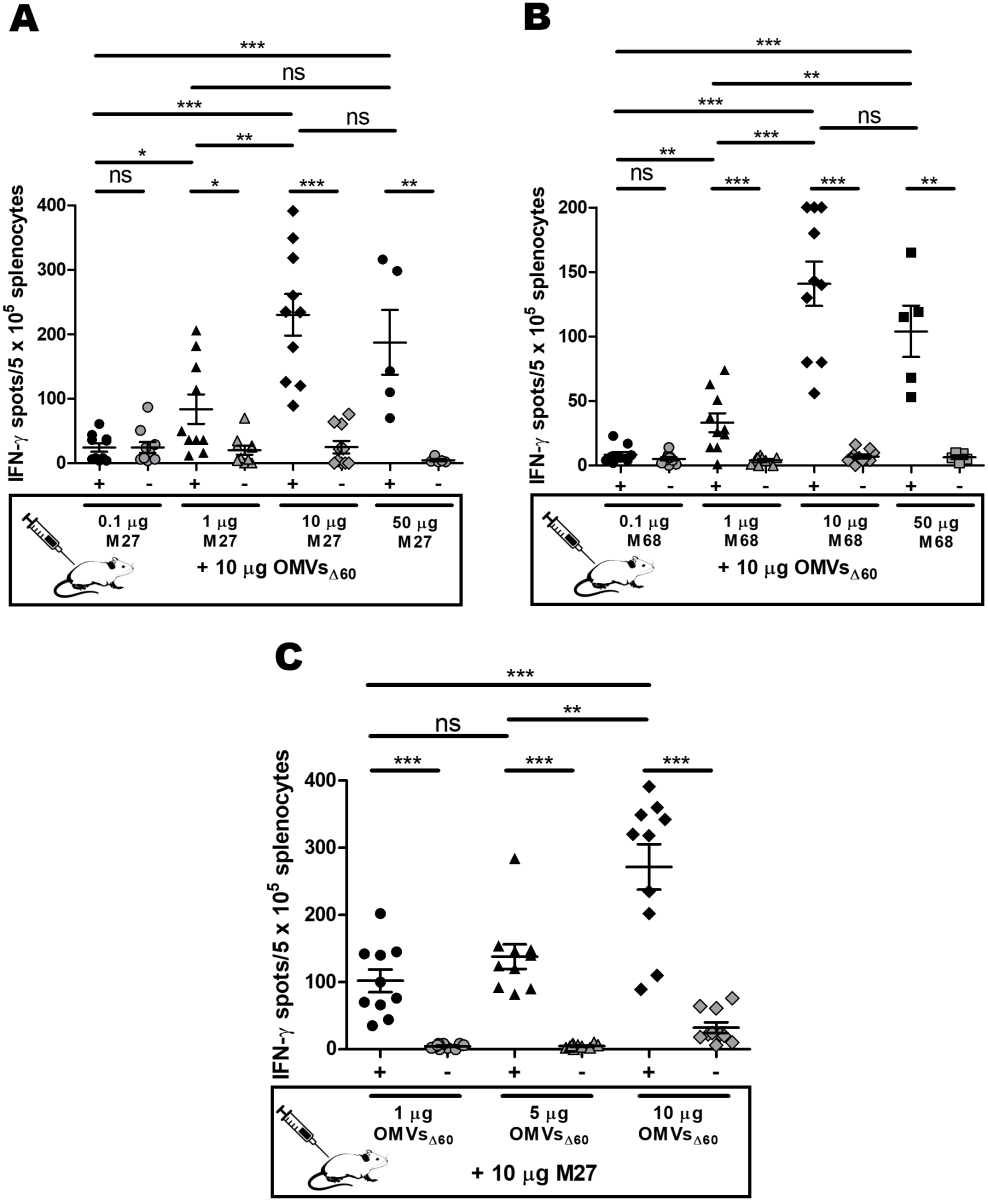
Influence of the peptide epitope and OMV amount on T-cell frequencies induced by vaccination; 0.1, 1, 10, or 50 µg of epitope peptide [M27 **(A)** and M68 **(B)**] was adsorbed to 10 µg of OMVs_Δ60_, and the mixtures were injected i.p. in BALB/c mice on day 0 and day 7. Splenocytes were stimulated on day 12 with either 5 µg/mL of vaccination peptide epitope (black) or 5 µg/mL of an unrelated peptide as a control (gray). **(C)** Three groups of mice were i.p. immunized with 10 µg of M27 peptide plus 1, 5, or 10 µg of OMVs_Δ60_. Splenocytes harvested on day 12 were stimulated with either 5 µg/mL of the M27 peptide (black) or 5 µg/mL of an unrelated peptide as a control (gray). Statistical significance was calculated using an unpaired, two-tailed Student’s *t*-test. ns, not significant; *P < 0.1; **P < 0.01; ***P < 0.001.

Having confirmed that *E. coli* OMVs_Δ60_ promote the production of IFN-γ^+^ T cells when combined with a five-fold higher amount (w/w) of synthetic peptides corresponding to CD4^+^ T-cell epitopes, we analyzed how the peptide concentration affects the frequency of the epitope-specific T cells. Different amounts of M27 and M68 peptides, ranging from 0.1 to 50 µg, were mixed with 10 µg of OMVs_Δ60_ (in 200 µL of PBS), and after two sessions of i.p. immunization, the frequencies of the epitope-specific T cells were measured in the spleens of the vaccinated mice. As shown in **Figures 3A, B**, epitope-specific IFN-γ^+^ T cells were detected at peptide quantities ≥1 µg. The frequencies appeared to reach a plateau with 10 µg of peptides, and increasing the dose to 50 µg yielded no further improvement.

Next, we investigated the influence of the quantity of OMVs on T-cell frequencies. In this experiment, we used the optimal dose of peptides previously determined (10 µg), and we progressively reduced the amount of OMVs starting from 10 µg. More specifically, 10 µg of M27 was mixed with 1, 5, or 10 µg of OMVs_Δ60_ in 200 µL of PBS; mice were immunized i.p. twice, 7 days apart, and epitope-specific T cells were determined on day 12 by the ELISpot assay. As shown in **Figure 3C**, 1 µg of OMVs was sufficient to elicit good frequencies of epitope-specific T cells.

Altogether, the data reported in **Figure 3** lead to the following considerations. First, appreciable amounts of epitope-specific IFN-γ^+^ T cells can be detected with peptide quantities higher than 1 µg, corresponding to 10% of the total OMV proteins present in each immunization dose (w/w). Second, the 10-µg peptide + 10-µg OMVs_Δ60_ mixture appeared to be the optimal condition to elicit epitope-specific IFN-γ^+^ T cells, at least in the case of the tested epitopes. Third, considering that immunization with 10 µg of OMVs_Δ60_ elicits measurable quantities of epitope-specific IFN-γ^+^ T cells (>0.01% of the total splenocytes), only if the epitope concentration is at least 10% of the total OMV proteins, most, if not all, MHC II-restricted epitopes embedded in OMV endogenous proteins are not expected to induce IFN-γ-^+^ T cells at frequencies detectable using ELISpot analysis. Even a putative 15-amino-acid-long MHC II epitope present in the highly expressed OmpF membrane protein (OmpF is a 362-amino-acid protein representing 40% of the total OMVs_Δ60_ proteins) would hardly induce detectable levels of IFN-γ^+^ CD4^+^ T cells, considering that its concentration would be less than 2% of the total OMV proteins.

To experimentally support this latter consideration, we fused the MHC II epitopes M27 and M68 to the C-terminus of the 281-amino-acid-long FhuD2 protein, a protein that efficiently compartmentalizes in the OMVs and has been extensively used as a carrier to engineer OMVs with heterologous protein domains/epitopes (32). When expressed in *E. coli* BL21(DE3)*Δ60*, the FhuD2-M27 and FhuD2-M68 fusions represented approximately 25% of the total OMV proteins (**Supplementary Figure S1A**), with the two epitopes corresponding to approximately 2.5% of the total OMV protein content (10% of the fusions). The engineered OMVs (10 µg) were used to i.p. immunize BALB/c mice, and the presence of M27- and M68-specific IFN-γ^+^ T cells was analyzed by ELISpot. As expected, no appreciable levels of epitope-specific IFN-γ^+^ CD4^+^ T cells could be detected (**Supplementary Figures S1B, C**).

### Epitope-specific CD8^+^ T cells induced by OMVs_Δ60_

The capacity of OMVs to promote the production of cytotoxic CD8^+^ T cells specific for OMV-associated MHC I epitopes has been repeatedly documented (15, 27, 28, 40). However, similar to OMV-induced CD4^+^ T cells, data correlating epitope quantities with T-cell frequencies are missing.

**Figure 4:**
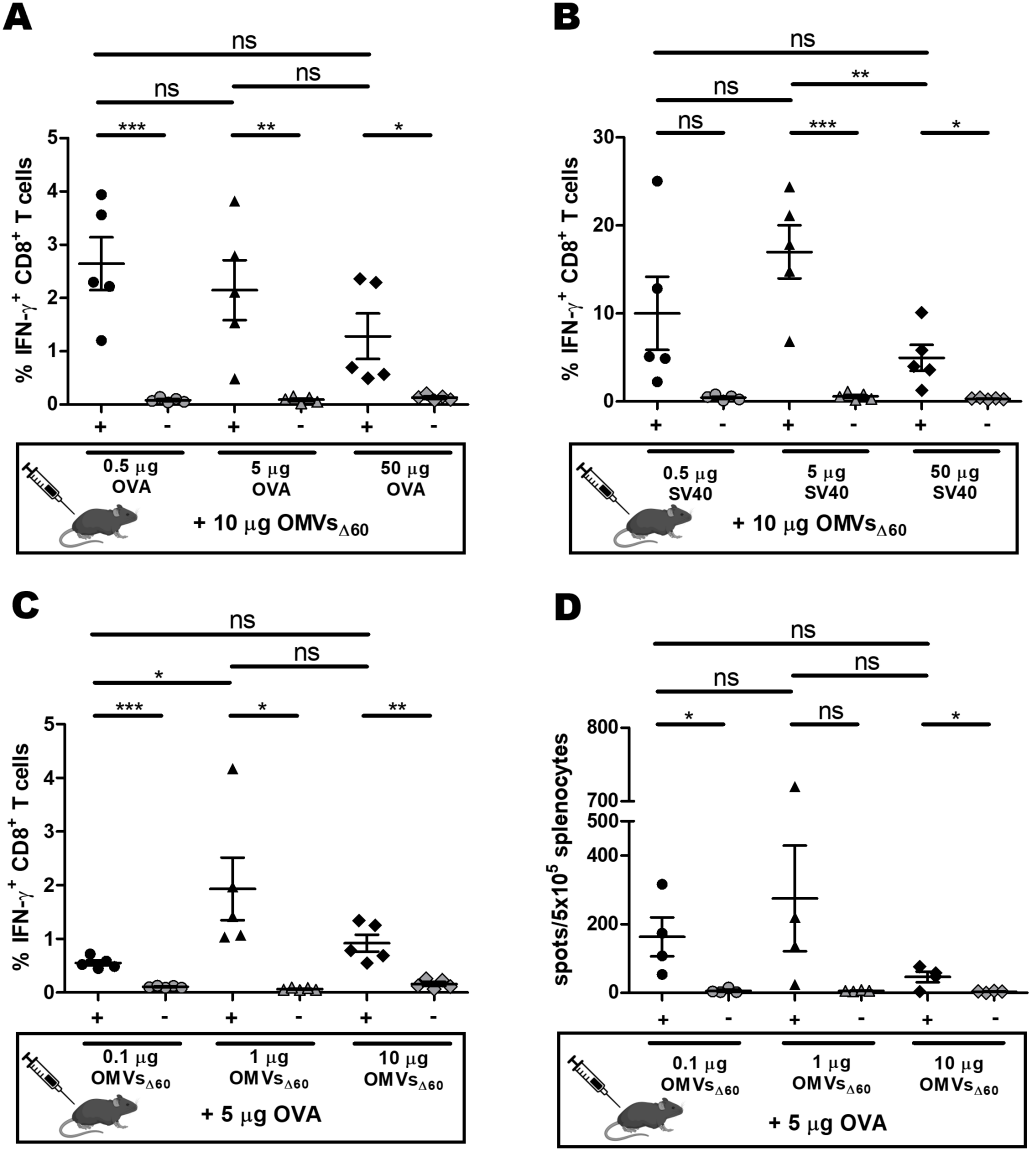
Analysis of epitope-specific T cells as a function of MHC I-restricted epitope and OMV concentrations. C57BL/6 mice were injected s.c. on days 0 and 7, and spleens were analyzed on day 12. Splenocytes were stimulated with either 5 µg/mL of the vaccination peptide (black) or 5 µg/mL of an unrelated peptide as a control (gray). **(A, B)** Three groups of mice were vaccinated with 10 µg of OMVs_Δ60_ mixed with 0.5, 5, or 50 µg of OVA **(A)** or SV40 **(B)** peptide epitope, and the frequencies of epitope-specific CD8 T-cell frequencies were analyzed by flow cytometry. **(C, D)** Three groups of mice were immunized with 5 µg of OVA peptide plus 0.1, 1, or 10 µg of OMVs_Δ60_, and the frequencies of epitope-specific CD8^+^ T-cell frequencies were analyzed by flow cytometry **(C)** or ELISpot assay **(D)**. Statistical significance was calculated using an unpaired, two-tailed Student’s *t*-test. ns, not significant; *P < 0.1; **P < 0.01; ***P < 0.001.

To address this question, we analyzed the effect of epitope concentration on cytotoxic T-cell production by immunizing mice with OMVs decorated with two different MHC I-restricted epitopes: OVA_257–264_ and SV40 IV_404–411_ C411L. We had previously shown that immunization of C57BL/6 mice with mixtures of 10 µg of OMVs_Δ60_ + 5 µg of either OVA or SV40 synthetic peptides elicited epitope-specific CD8^+^ T cells at frequencies between 5% and 10% of the total IFN-γ^+^ CD8^+^ T cells (34). Based on these results, using 10 µg of OMVs_Δ60_ as a fixed dose, we immunized groups of four or five mice each with three different amounts (0.5, 5, and 50 µg) of either OVA or SV40 peptides, and we analyzed the T-cell frequencies in the splenocytes. The experiment was repeated twice: in the first set, the T-cell frequencies were analyzed by flow cytometry, while in the second set, the frequencies were determined using the ELISpot assay. As shown in **Figures 4A–D**, 0.5 µg of peptides were sufficient to elicit epitope-specific T cells, and the frequencies did not substantially vary with different peptide concentrations. Although not tested in these experiments, the plateau level of T cells observed at OVA peptide concentrations above 0.5 µg suggested that even a lower amount of MHC I epitopes would be sufficient to induce measurable quantities of epitope-specific CD8^+^ T cells.

In conclusion, our data confirm that OMVs mixed with MHC I-restricted epitopes elicit epitope-specific CD8^+^ T cells and that formulations containing epitope quantities as low as 0.5 µg, and probably lower, can elicit high frequencies of epitope-specific CD8^+^ T cells.

In contrast to what was observed with MHC II-restricted epitopes, the results described above suggest that MHC I-restricted epitopes present in OMV proteins should induce frequencies of epitope-specific CD8^+^ T cells high enough to be measurable by flow cytometry and/or ELISpot. Even if 0.5 µg was the minimal amount of epitope necessary to elicit measurable CD8^+^ T cells, this amount could be reached by any MHC I epitope present in 20 to 40 kDa of OMV proteins expressed at a level of approximately 10% to 20% of the total OMV proteins.

**Figure 5:**
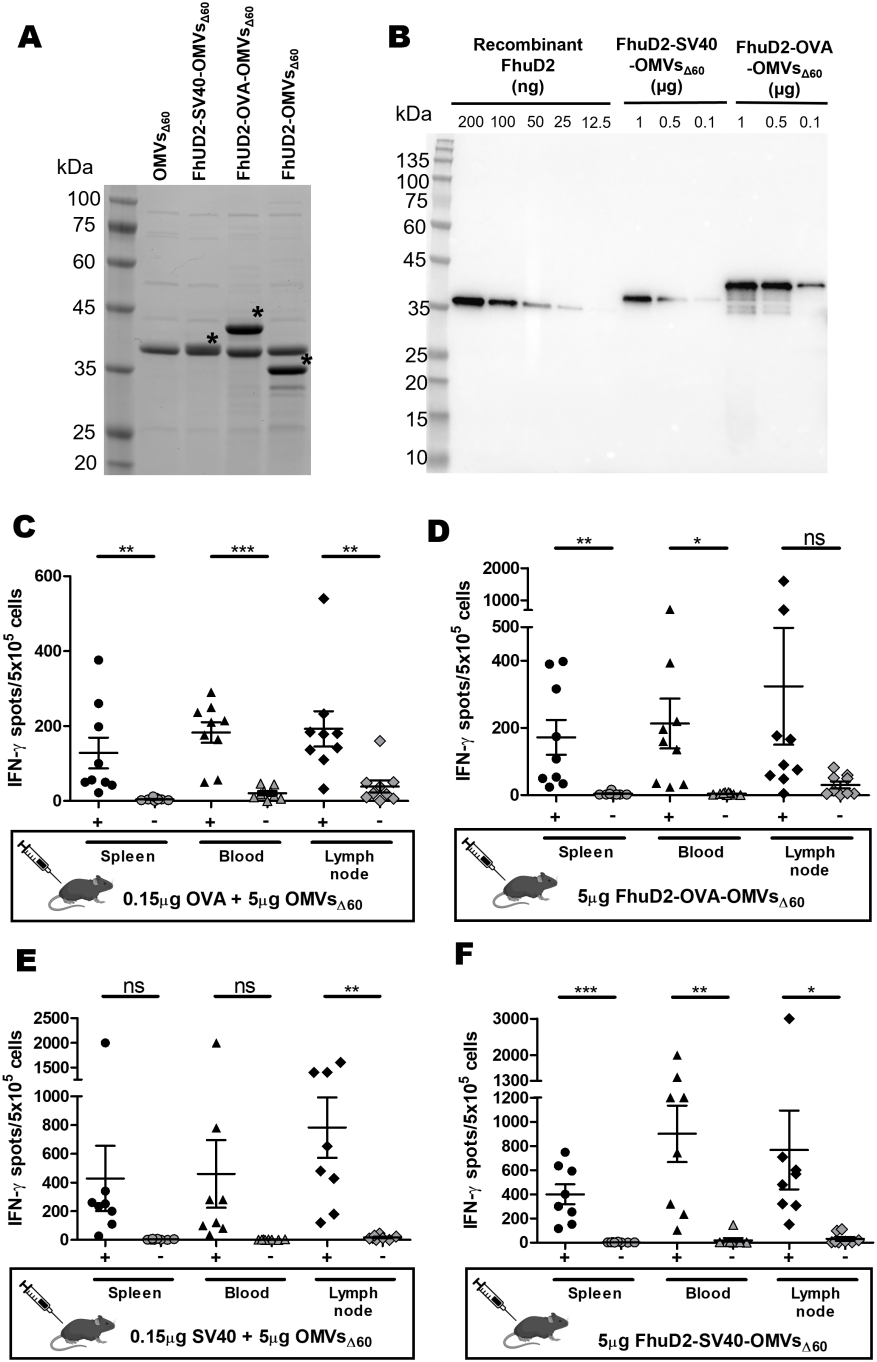
Epitope-specific IFN-γ^+^ CD8^+^ T cells elicited by immunizations with OMVs_Δ60_ mixed with MHC I-restricted synthetic epitopes and with OMVs_Δ60_ engineered with the MHC I-restricted epitope IFN-γ^+^ CD8^+^ T cells. **(A)** SDS-PAGE analysis of OMVs_Δ60_ engineered with either SV40 or OVA epitopes fused to the C-terminus of FhuD2 carrier protein. Purified OMVs (20 µg) were loaded on the gel, and protein species were visualized by Coomassie Blue staining. The asterisks indicate the bands corresponding to FhuD2-SV40 fusion (lane 3), FhuD2-OVA fusion (lane 4), and FhuD2 carrier protein (lane 5). **(B)** Semi-quantification of the fusion proteins expressed in OMVs by Western blot analysis. Escalating doses of purified recombinant FhuD2 (standard), FhuD2-SV40-OMVs_Δ60_, and FhuD2-OVA-OMVs_Δ60_ were separated on a polyacrylamide gel, transferred to a nitrocellulose membrane, and visualized using anti-FhuD2 antibodies. Visual comparison of the band intensities between the standard and the OMV samples was used to quantify the amount of fusion proteins in OMVs (see text for details). **(C–F)** C57BL/6 mice were s.c. immunized two times on day 0 and day 7 with 5 µg of FhuD2-OVA-OMVs_Δ60_ **(C)**, 5 µg of OMVs_Δ60_ adsorbed with 0.15 µg of OVA peptide **(D)**, 5 µg of FhuD2-SV40-OMVs_Δ60_ **(E)**, or 5 µg of OMVs_Δ60_ adsorbed with 0.15 µg of SV40 peptide **(F)**. After 5 days from the second immunization, splenocytes/PBMCs/draining lymph nodes were collected from each mouse, and peptide‐specific IFN-γ^+^ CD8^+^ T cells were analyzed by ELISpot, after stimulation with 5 µg/mL of the vaccination peptide (black) or 5 µg/mL of an unrelated peptide as a control (gray). Statistical significance was calculated using an unpaired, two-tailed Student’s *t*-test. ns, not significant; *P < 0.1; **P < 0.01; ***P < 0.001.

To test this, we engineered OMVs_Δ60_ with OVA and SV40 epitopes. In particular, the synthetic DNA encoding three copies of the OVA epitopes, each copy separated from each other by a glycine-glycine spacer, was fused to a plasmid carrying the FhuD2 gene, thus generating the recombinant plasmid pET-FhuD2-OVAx3 (see *Materials and methods*, **Table 1**). The pET-FhuD2-SV40x1 plasmid was generated in the same manner but with only one copy of the epitope (see *Materials and methods*, **Table 1**). After transformation in *E. coli* BL21(DE3)*Δ60*, OMVs were purified from the culture supernatants (FhuD2-OVA-OMVs_Δ60_ and FhuD2-SV40-OMVs_Δ60_). As shown in **Figure 5A**, the FhuD2-OVAx3 and FhuD2-SV40x1 fusions accumulated in the OMVs at approximately 40% and 10%, respectively, of the total OMV proteins, as observed by semi-quantitative Western blot analysis (**Figure 5B**). Considering that the three copies of the OVA epitope account for 10% of the fusion protein (24 amino acids out of 300) and the SV40 epitope accounts for 3% of the total fusion protein (8 amino acids out of 280), 10 µg of engineered OMVs carry approximately 0.40 µg of OVA epitope and 0.03 µg of SV40 epitope. Next, four groups of C57BL/6 mice were s.c. immunized with four different formulations: 5 µg of OMVs_Δ60_ + 0.15 µg of OVA peptide, 5 µg of OMVs_Δ60_ + 0.15 µg of SV40 peptide, 5 µg of FhuD2-OVA-OMVs_Δ60_, and 5 µg of FhuD2-SV40-OMVs_Δ60_. Animals were given two doses of vaccines on days 0 and 7, and on day 12, epitope-specific CD8^+^ T cells were analyzed in the spleen, blood, and draining lymph node by the ELISpot assay. The experiment was repeated twice to obtain T-cell frequency data from a total of nine mice per group. First, 0.15 µg of both OVA and SV40 epitope peptides mixed to OMVs_Δ60_ were sufficient to elicit appreciable frequencies of epitope-specific T cells, which could be found in all three compartments (**Figures 5C, E**). Second, 5 µg of FhuD2-OVA-OMVs_Δ60_ and FhuD2-SV40-OMVs_Δ60_, which carry approximately 0.2 µg of OVA and 15 ng of SV40 epitopes, respectively, elicited epitope-specific T cells whose quantities were not dissimilar from those found in mice immunized with the peptide–OMV mixtures (**Figures 5D, F**).

**Table 1:** Nucleotide sequences of the synthetic genes encoding the MHC I and MHC II epitopes.

OVAx3	5′-CAGCTGGAAAGCATTATTAACTTTGAAAAACTGACCGAAGGTGGTCAGCTGGAAAGCATTATTAACTTTGAAAAACTGACCGAAGGTGGTCAGCTGGAAAGCATCATCAACTTCGAAAAACTGACCGAA-3′
SV40x1	5′-GATAGCGTGGTGTATGATTTTCTGAAACTGATGGTG-3′
M27	5′-GAGCATATTCATCGTGCTGGTGGACTTTTTGTGGCTGACGCAATTCAAGTAGGATTTGGACGCATCGGTAAGCATTTCTGG-3′
M68	5′-GTAACAAGCATCCCATCCGTCTCTAATGCTCTGAATTGGAAAGAATTTTCGTTTATTCAGAGTACCTTGGGCTACGTGGCC-3′

## Discussion

OMVs are gaining increasing interest in vaccine applications, as indicated by the 7,063 patents and 920 papers annotated, under the string search “bacterial outer membrane vesicles AND vaccines” (as of May 2025), in the patent and publication databases Espacenet and PubMed, respectively.

Despite this biotechnological interest, certain aspects of OMV-induced immunity are still poorly understood. Among them is the role played by OMV-associated proteins in the overall immune response and how these proteins interfere or cooperate with each other in determining the quality and quantity of the OMV humoral and cell-mediated immunity.

**Figure 6:**
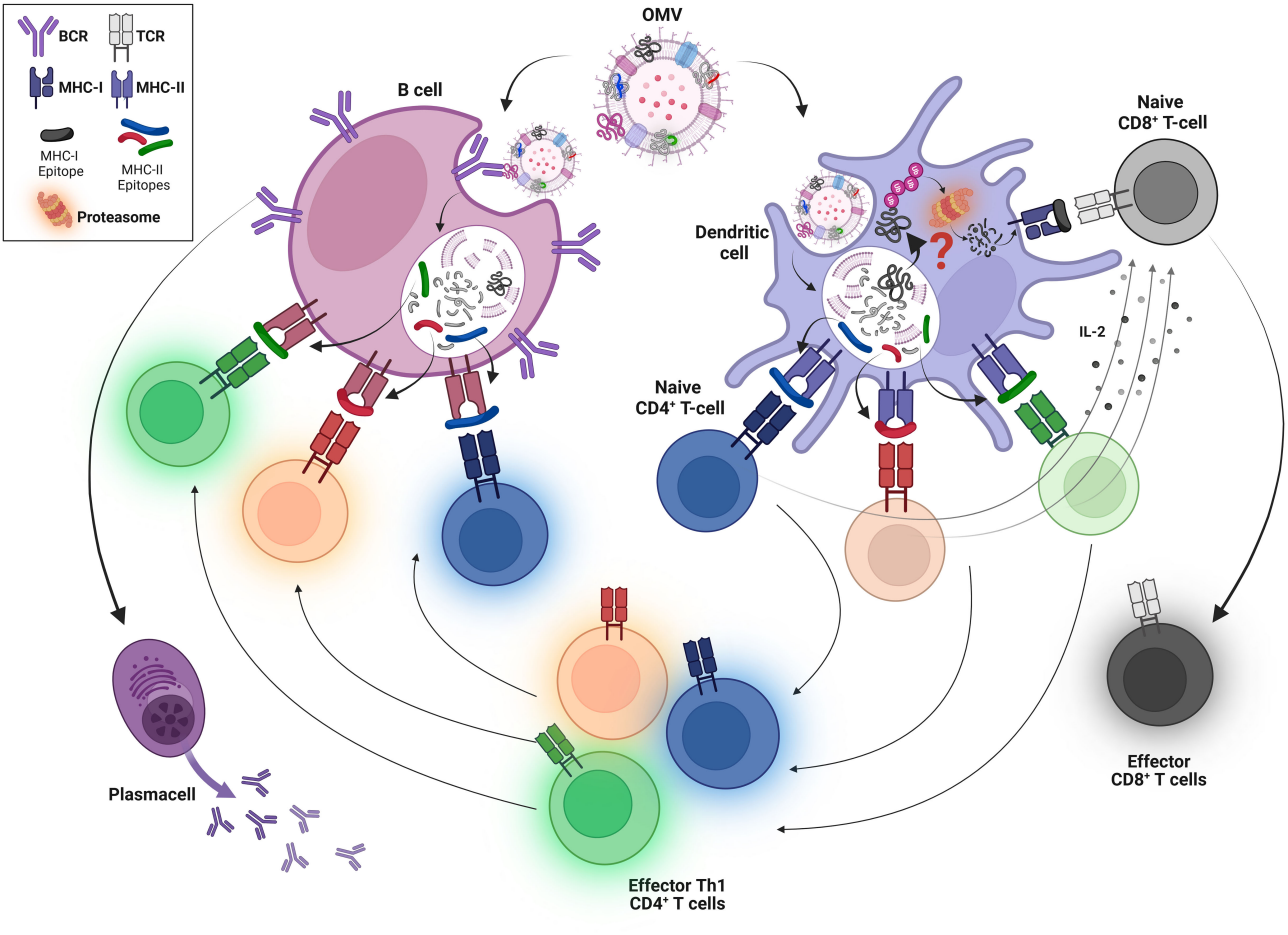
Schematic representation of the predicted mechanisms of OMV-induced cell-mediated and humoral immune responses. OMVs are internalized by DCs, which process and present OMV-derived CD4^+^ T-cell epitopes on MHC class II molecules to naive CD4^+^ T cells. This leads to the production of a large polyclonal population of effector INF-γ^+^, CD4^+^ T cells. At the same time, OMVs/OMV antigens, through a still unclear mechanism (?), exit the endosomal compartment where they are degraded by the proteasome, generating peptides that are loaded onto MHC class I molecules. This allows the production of a high number of epitope-specific CD8^+^ T cells, thanks to the stimulation received from the polyclonal population of OMV-derived INF-γ^+^, CD4^+^ T cells (shown by arrows). In parallel, OMVs are internalized by B cells through BCR-mediated recognition of OMV-associated antigens. This uptake enables B cells to process and present OMV-associated MHC II epitopes, which are recognized by the polyclonal population of INF-γ+, CD4^+^ T cells elicited by DCs after OMV internalization. This allows a robust production of antibodies even against proteins that are poorly expressed in OMVs.

In an attempt to shed light on this important aspect, we recently investigated how the level of expression of OMV proteins affects their capacity to induce specific antibodies. In other words, we investigated the minimum quantity of a specific OMV-associated protein necessary and sufficient to elicit specific antibodies upon OMV immunization. We found that OMV immunization promotes the production of elevated IgG titers, predominantly IgG2a, against those proteins present in the OMVs at a concentration ≥0.5% of the total OMV protein content (w/w). It was observed that 50 to 100 ng of any OMV-associated protein is sufficient to induce antibody responses when a vaccine dose of 10 µg of OMVs is used (35). Such remarkable immunogenicity can be explained assuming that each antigen-specific B cell can receive support from the polyclonal population of CD4^+^ effector T cells induced by the numerous OMV-associated MHC II epitopes (**Figure 6**). From a biotechnological standpoint, this information strongly supports the use of engineered OMVs in the design of those vaccines whose efficacy is based on the elicitation of humoral immunity.

The model described above, showing that the abundance of OMV-associated MHC II epitopes leads to the production of a broad population of CD4^+^ T-cell clones, also predicts that each T-cell clone is represented by a relatively low number of cells. In other words, the model predicts that to obtain high CD4^+^ T-cell frequencies against a specific epitope, the epitope concentration should overwhelm the concentration of all the other OMV-associated MHC II epitopes. Consistent with this model, in this work, we found that to detect appreciable levels of IFN-γ^+^ CD4^+^ T cells specific for a selected epitope, the epitope concentrations have to be higher than 10% of the total OMV proteins (w/w). This result implies that even the epitopes present in the most abundant OMV proteins, which can account for up to 40% of the total OMV proteins (this is, for instance, the case of OmpF in OMV_Δ60_), would hardly induce frequencies of IFN-γ^+^ CD4^+^ T cells ≥0.2% of the total CD4^+^ T-cell population, the threshold T-cell frequency usually detectable by flow cytometry and ELISpot. This information is relevant in the design of effective vaccines, which need to elicit high frequencies of epitope-specific IFN-γ^+^ CD4^+^ T cells. For such vaccines, the use of the OMV platform offers the advantage to skew the immune response toward a Th1 profile; however, it requires the external addition of recombinant or synthetic MHC II epitopes to ensure a sufficiently high amount of epitope.

As far as the elicitation of CD8^+^ T cells by OMV-associated MHC I epitope is concerned, we tested the influence of epitope concentration on the frequencies of epitope-specific CD8+ T cells using the two widely used OVA and SV40 MHC I-restricted epitopes. OVA_257_–SIINFEKL_-264_ (OVA) is a CD8^+^ T-cell epitope derived from chicken ovalbumin protein and specific for the MHC I H-2 Kb allotype. OVA is one of the most studied CD8^+^ T-cell epitopes both *in vitro* and *in vivo* due to its high immunogenicity and the availability of specific reagents. Immunization of C57BL/6 mice with OVA synthetic peptide formulated with different adjuvants elicits high frequencies of OVA-specific T cells (41, 42). SV40 IV_404_-VVYDFLKL_-411_ C411L (SV40) is another well-studied MHC I H2-Kb-restricted epitope derived from the Simian virus 40 (SV40) large tumor antigen. C57BL/6 mice immunized with B6/T116A1 cells expressing SV40 epitopes elicit frequencies of approximately 8% of SV40-specific IFN-γ^+^ CD8^+^ T cells (43, 44). The data presented in this study show that even epitope concentrations below 1% of the total of OMV proteins are sufficient to elicit good frequencies of epitope-specific CD8^+^ T cells (**Figure 5**). Such amounts can be reached by either mixing synthetic peptides with OMVs or using appropriate engineering strategies. In this work, we fused one or more copies of the MHC I model epitopes OVA and SV40 to the C-terminus of the FhuD2 carrier protein. We found that immunization with engineered OMVs in which the OVA and the SV40 epitopes represent approximately 0.4% and 0.05% of the total OMV protein content, respectively, elicits excellent CD8^+^ T-cell frequencies. The efficiency with which the OMV-associated MHC I epitopes induce CD8^+^ T cells is consistent with the mechanism of CD8^+^ T-cell activation involving a polyclonal CD4^+^ T-cell help response (**Figure 6**). According to this mechanism, the OMVs carrying a specific MHC I epitope (either engineered or adsorbed to the OMV surface) are taken up by the APCs, which simultaneously present both the MHC I epitope and the OMV-associated MHC II epitope on their surface (**Figure 6**). This would allow the activation of the epitope-specific CD8^+^ T cells by a variety of CD4^+^ T helper cells.

We believe that our data are relevant for a better understanding of OMV-induced cellular immunity and for designing effective OMV-based vaccines.

A few important questions still remain to be addressed. First of all, our studies were carried out using OMVs_Δ60_, which are vesicles released by the *E. coli* BL21(DE3)*Δ60* derivative created in our laboratories and deprived of 59 OMV-associated proteins. Whether the conditions used to induce T cells with OMVs_Δ60_ apply to vesicles derived from other *E. coli* strains and other Gram-negative species remains to be investigated. Second, the fate of OMVs after being taken up by APCs and how OMV proteins are processed and presented by MHC I and MHC II molecules are unknown. In particular, after endocytosis and OMV degradation, a still open question is whether all OMV-associated MHC II epitopes have a similar chance to compete with each other for binding to MHC II molecules or if some epitopes, belonging to either the membrane or the luminal compartment, have a preferential path to MHC II presentation. Addressing this question is important to understand the extent of polyclonality of CD4^+^ T-cell responses and to predict which OMV proteins are likely to elicit specific CD4^+^ T cells. Third, the mechanism through which OMV proteins reach the cytoplasm and get processed by the proteasome to ultimately generate peptides presented on MHC I molecules is still unclear. One proposed interesting model is that in the early endosomes, OMVs preserve their integrity, but their surface-exposed proteins are “shaved” by endosomal proteases, and the released products exit the early endosomes and enter the MHC I presentation pathway (45). This model is consistent with the data presented here showing that OVA and SV40-specific CD8^+^ T cells are elicited when the epitopes are fused to the surface-exposed FhuD2 carrier protein (US patent 12054521B2) and with previously published data reporting that only the MHC I epitopes localized on the surface of OMVs can induce CD8^+^ T cells (15), while luminal proteins mostly induce CD4^+^ T cells (23). An alternative model would envisage that intact OMVs can somehow exit the early endosomes and are degraded in the cytoplasm, and the OMV proteins are digested by the proteasome. This model would not exclude that luminal OMV proteins could induce CD8^+^ T cells. Indeed, in the preliminary experiments, we expressed the MHC I OVA epitope fused to MBP. Even though fusion was compartmentalized in the lumen of the OMVs, immunization with the engineered OMVs elicited OVA-specific CD8^+^ T cells (Tomasi et al., unpublished).

Fourth, in our work, we focused on IFN-γ^+^ CD4^+^ T cells, considering that our OMVs predominantly elicit a Th1-skewed immune response (34). However, it has been well documented that OMVs can also induce Th17 responses. Therefore, the frequencies of epitope-specific T cells could be higher and consequently measurable if both IFN-γ^+^ and IL-17a-positive CD4^+^ T cells were analyzed. This aspect remains to be investigated.

Finally, as pointed out before, based on the high number of predicted OMV-associated MHC II epitopes, we assume that the OMV-induced CD4^+^ T-cell population is highly polyclonal. However, the extent of CD4^+^ T-cell polyclonality has not been characterized. One way to elucidate this important aspect of OMV immunity would be to isolate the T-cell population after OMV immunization and to run single-cell TRC sequencing. Experiments along this line are in progress.

## Materials and methods

### Animal studies

Six- to 8-week-old female BALB/c and C57BL/6 mice were purchased from the Charles River Laboratories. All mice were treated in accordance with the Animal Ethics Committee of the University of Trento and the Italian Ministry of Health. Animal experiments were carried out in accordance with experimental protocols reviewed and approved (1060/2016-PR and 1153/2020-PR) by the Animal Ethics Committees of the University of Trento (Trento, Italy), Toscana Life Sciences Foundation (Siena, Italy), and the Italian Ministry of Health. Mice were monitored once per day to evaluate early signs of pain and distress, such as respiration rate, posture, and loss of weight (more than 20%) according to humane endpoints. Mice showing such conditions were anesthetized and subsequently sacrificed in accordance with experimental protocols.

For immunogenicity analyses, animals were intraperitoneally or subcutaneously immunized two (days 0 and 7) or three (days 0, 7, and 14) times with different amounts of OMVs_Δ60_ and peptides in a final volume of 200 µL/dose for i.p. or 100 µL/dose for s.c. immunizations, formulated in phosphate-buffered saline (PBS) (Gibco, Thermo Fisher Scientific, MA, USA). Spleen, blood, or lymph nodes were analyzed 5 days after the last immunization.

### Bacterial strains and culture conditions

Plasmid assembly using the polymerase incomplete primer extension (PIPE) method (46) was carried out in *E. coli* HK-100 strain. *E. coli* was routinely grown in LB broth medium (25 g/L, Sigma-Aldrich, St. Louis, MO, USA) at 30°C and/or 37°C and 180 rpm. When required, ampicillin (Amp) was added to a final concentration of 100 µg/mL. Stock preparations of strains in LB and 20% glycerol were stored at −80°C. Each bacterial manipulation was started from an o/n culture inoculum of a frozen stock or of a single colony from an LB plate. Recombinant clones were grown at 30°C and 180 rpm in LB medium (starting OD_600_ = 0.05), and when the cultures reached an OD_600_ of 0.5–0.7, protein expression, when needed, was induced by the addition of 0.1 mM IPTG (Sigma-Aldrich, St. Louis, MO, USA). OMVs were purified from the *E. coli* BL21(DE3)*Δ60* strain (34) as previously described (27, 28, 32, 47).

### Synthetic peptides

The synthetic peptides used in the study were purchased from GenScript Biotech (Piscataway, New Jersey, USA). The amino acid sequences of the peptides are reported in **Table 2**.

**Table 2:** Synthetic peptides used in this study.

Peptide name	Amino acid sequence
OVAx3	QLESIINFEKLTEGGQLESIINFEKLTEGGQLESIINFEKLTE
SV40x1	DSVVYDFLKLMV
M03	DKPLRRNNSYTSYIMAICGMPLDSFRA
M20	PLLPFYPPDEALEIGLELNSSALPPTE
M27	EHIHRAGGLFVADAIQVGFGRIGKHFW
M68	VTSIPSVSNALNWKEFSFIQSTLGYVA

The CD8 epitope sequences are underlined.

### Engineering of OMVs with MHC I and MHC II epitopes

The pET-FhuD2-OVAx3 plasmid was assembled using the PIPE method (46). Briefly, the pET21-FhuD2 plasmid carrying the *Staphylococcus aureus* ferric hydroxamate receptor 2 (FhuD2) was linearized by PCR, using FhuD2-v-R and pET-V-F primers (**Table 2**). In parallel, the synthetic gene encoding three copies of the OVA_257–264_ epitope with its flanking sequences separated by a glycine-glycine flexible spacer was purchased from GeneArt^®^ Gene Synthesis (Thermo Fisher Scientific, Waltham, MA, USA). The full nucleotide and amino acid sequences of the construct are provided in **Tables 1** and **3**, respectively. The OVA construct was amplified with the OVA_FhuD2-F and OVA_FhuD2-R primers (**Table 2**). Finally, PCR products were mixed together and used to transform *E. coli* HK100 competent cells, obtaining the pET-FhuD2-OVAx3 plasmid.

**Table 3:** Synthetic oligonucleotides used for cloning the OVA synthetic gene.

Oligo name	Sequence
OVA_FhuD2-F	5′-TAATTAAAGCTGCAAAACAGCTGGAAAGCATTATTAACTTTGAAAAAC-3′
OVA_FhuD2-R	5′-TGGTGATGGTGATGTTATTCGGTCAGTTTTTCGAAGTTGATGATGCTTTC-3′
FhuD2-Rev	5′-TTTTGCAGCTTTAATTAATTTTTCTTTTAAATCTTTACGC-3′
pET_F	5′-TAACATCACCATCACCATCACGATTACAAAGA-3′

The pET-FhuD2-SV40x1, pET-FhuD2-M27, and pET-FhuD2-M27 plasmids were assembled with restriction enzymes. pET21-FhuD2 was digested with *Bam*HI and *Xho*I restriction enzymes at the end of the FhuD2 gene. The synthetic gene encoding one copy of the SV40 IV_404–411_ C411L epitope with its flanking sequences was generated by annealing two oligonucleotides purchased from Eurofins Genomics (Ebersberg, Germany, EU). The synthetic genes encoding one copy of M27 and M68 epitopes were generated by annealing two oligonucleotides purchased from Eurofins Genomics (Ebersberg, Germany, EU). The full nucleotide and amino acid sequences of the final constructs are shown in **Tables 1** and **3**, respectively. SV40, M27, and M68 synthetic genes were digested with *Bam*HI and *Xho*I restriction enzymes and ligated to the digested pET21-FhuD2 plasmid. Ligation products were then used to transform *E. coli* HK100 competent cells, obtaining the pET-FhuD2-SV40x1, pET-FhuD2-M27, and pET-FhuD2-M68 plasmids.

To confirm the correct gene fusions, plasmids were sequenced (Eurofins Genomics, Ebersberg, Germany, EU). The *E. coli* BL21(DE3)*Δ60* strain was transformed with the pET-FhuD2-OVAx3, pET-FhuD2-SV40x1, pET-FhuD2-M27, and pET-FhuD2-M68 plasmids, and the derived recombinant strains were used for the production of engineered FhuD2-OVA OMVs_Δ60_, FhuD2-SV40 OMVs_Δ60_, FhuD2-M27 OMVs_Δ60_, and FhuD2-M68 OMVs_Δ60_, respectively.

### SDS-PAGE and Western blot

OMV protein content was analyzed by the DC protein assay (Bio-Rad, Hercules, CA, USA). Ten micrograms of OMVs (protein content) or recombinant proteins were resuspended in sodium dodecyl sulfate-polyacrylamide gel electrophoresis (SDS-PAGE) Laemmli buffer and heated at 100°C for 10 min. Proteins were separated using Any kD™ Criterion™ TGX Stain-Free™ Protein Gel (Bio-Rad, Hercules, CA, USA) in Tris-glycine buffer (Bio-Rad, Hercules, CA, USA). Finally, proteins were revealed by Coomassie Blue staining. For Western blot analysis, proteins separated by SDS-PAGE were subsequently transferred onto nitrocellulose membrane (iBlot™ Transfer Stack, nitrocellulose, Thermo Fisher Scientific) with iBlot^®^ Dry Blotting System (Thermo Fisher Scientific). The membranes were blocked at room temperature (RT) for 30 min by agitation in blocking solution [10% skimmed milk and 0.05% Tween 20 dissolved in PBS (PBST)]. Rabbit polyclonal anti-FhuD2 antibodies (pAbs) were incubated for 1 h at RT or o/n at 4°C. The pAbs were custom-made by GenScript and used at 0.5 µg/mL concentration in 1% skimmed milk-PBST. After three washing steps of 5 min in PBST, the membranes were incubated in a 1:2,000 dilution of peroxidase-conjugated anti-rabbit total IgGs (Dako, Glostrup, Denmark) for 1 h in 1% skimmed milk-PBST, and after two washing steps of 5 minutes in PBST and one washing step of 5 min in PBS, antibody binding was detected using the SuperSignal West Pico chemiluminescent substrate (Thermo) and visualized with a ChemiDoc (Bio-Rad, Hercules, CA, USA) instrument.

### Splenocytes, PBMCs, and lymph node cells

Spleens and lymph nodes were homogenized, and splenocytes and lymph node cells were filtered using a 70-µm cell strainer (BD). Splenocytes and lymph node cells were resuspended at 2.5 × 10^6^ cells per mL in Roswell Park Memorial Institute (RPMI) medium (Gibco) + 10% heat-inactivated fetal bovine serum (FBS) (Gibco) and 2 mM of L-glutamine (Fisher Scientific) and 1× penicillin/streptomycin (EuroClone, Milan, Italy) (complete RPMI).

Blood was collected by cardiac puncture. Approximately 500 µL of blood was obtained from each BALB/c or C57BL/6 mouse to which 25 USP units/sample of heparin (Sigma-Aldrich, St. Louis, MO, USA) was added. The blood sample was diluted to 1:1 (volume/volume) with PBS and added to Ficoll-Paque PLUS (GE Healthcare, Chicago, IL, USA) in a 12-mm × 75-mm, 5-mL polystyrene-capped tube. After centrifugation at 400×*g* for 30 min with no brakes, the buffy coat of PBMCs was isolated with a Pasteur pipette. PBMCs were washed twice in PBS (300×*g* for 5 min) and resuspended in complete RPMI.

### IFN-γ ELISpot

ELISpot assay was performed according to the manufacturer’s instructions. ELISpot plates [Mouse IFN-γ ELISpot PLUS kit (ALP), Mabtech, Stockholm, Sweden] were washed four times with sterile PBS (200 µL/well). Subsequently, the plates were conditioned with complete RPMI (200 µL/well) and incubated for 30 min at room temperature. The medium was then removed, and 2.5 × 10^5^ cells resuspended in 100 µL of medium were added to each well. For stimulation, 100 µL of 5 µg/mL solution peptides were added. As positive and negative controls, cells were stimulated with phorbol 12-myristate 13-acetate (PMA, 0.5 µg/mL) and ionomycin (1 µg/mL) or with 5 µg/mL of an unrelated peptide, respectively. The plates were put in a 37°C humidified incubator with 5% CO_2_ and incubated for 12–18 h. The cells were then removed, and the plates were washed five times with 200 µL/well of PBS. The detection antibody (R4-6A2-biotin) was diluted to 1 µg/mL in PBS containing 0.5% fetal calf serum (PBS-0.5% FBS). 100 µL/well was added to each well, and the plates were incubated for 2 h at room temperature. After this step, the plates were washed five times with sterile PBS (200 µL/well). Streptavidin-ALP was diluted 1,000 times in PBS-0.5% FBS, and 100 µL/well was added for 1 h at room temperature. The plates were washed five times with 200 µL/well of PBS. The substrate solution (BCIP/NBT-plus; BCIP: 5-bromo-4-chloro-3-indolyl phosphate; NBT: nitro blue tetrazolium) was 0.5-µm filtered, and 100 µL was added to each well. Spots were developed for 15–20 min, and the reaction was stopped by washing in tap water. The spots were counted using a stereomicroscope. Graphs and statistical analyses (unpaired, two-tailed Student’s *t*-test) were performed with GraphPad Prism 5.03.

### Intracellular cytokine staining on splenocytes

Splenocytes were collected and stimulated with 5 µg/mL of OVA or SV40 peptide or with 5 µg/mL of an unrelated peptide as a negative control. As a positive control, cells were stimulated with 0.5 µg/mL of PMA and 1 µg/mL of ionomycin. After 2 h of stimulation at 37°C, GolgiStop (BD Bioscience, San Jose, CA, USA) was added to each well, and cells were incubated for 4 h at 37°C. After two washes with PBS, LIVE/DEAD™ Fixable Near-IR Dead cell staining reaction mixture (Thermo Fisher, Waltham, MA, USA) was added for 20 min at room temperature in the dark. After two washes with PBS and permeabilization and fixing with Cytofix/Cytoperm™ (BD Bioscience, San Jose, CA, USA) using the manufacturer’s protocol, splenocytes were stained with a mix of the following antibodies: anti-CD3-APC (BioLegend, San Diego, CA), anti-CD4-BV510 (BioLegend, San Diego, CA), anti-CD8-PE-CF594 (BD), and IFN-γ-BV785 (BioLegend, San Diego, CA). Samples were acquired on a BD LSRII FACS and analyzed using the FlowJo software. Graphs and statistical analyses (unpaired, two-tailed Student’s *t*-test) were performed with GraphPad Prism 5.03.

## Data Availability

The original contributions presented in the study are included in the article/**Supplementary Material**. Further inquiries can be directed to the corresponding author.

## Supplementary material

Supplementary Figure 1 Epitope-specific IFN-γ^+^ CD4^+^ T cells elicited by immunizations with OMVs_Δ60_ engineered with MHCII-restricted epitopes M27 and M68. **(A)** SDS-PAGE analysis of OMVs_Δ60_ engineered with M27 and M68 MHCII epitopes fused to the C-terminus of FhuD2 carrier protein (see Text for details). Purified OMVs (10 µg) were loaded on the gel and protein species were visualized by Coomassie Blue staining. The asterisks indicate the bands corresponding to the FhuD2-M27 fusion (lane 3), FhuD2-M68 fusion (lane 4) and to FhuD2 carrier protein (lane 5). Quantification of the fusion proteins expressed in OMVs was performed by densitometric analysis using Image Lab software (BioRad). **(B, C)** BALB/c C57BL/6 mice were i.p. immunized on day 0 and day 7 with 10 µg of OMVs_Δ60_ engineered with either FhuD2-M27-OMVs_Δ60_ **(B)** or 10 µg of FhuD2-M68-OMVs_Δ60_ **(C)** and after 5 days from the second immunization, splenocytes, were collected from each mouse and peptide‐specific IFN-γ^+^ CD4^+^ T cells were analyzed by ELISpot, after stimulation with the 5 µg/ml of peptide used in vaccination (black) or 5 µg/ml of an unrelated peptide (gray). Statistical significance was calculated using an unpaired, two-tailed Student’s t-test. ns, not significant, *P<0.1; **P<0.01; ***P<0.001.
