# Supplementary figures and images for: Investigation on antigen-specific T-cell responses induced by outer membrane vesicles from Escherichia coli Δ60 strain
Source: Front Immunol. 2025 Oct 14;16:1633961. doi: 10.3389/fimmu.2025.1633961 (PMC12558846; doi:10.3389/fimmu.2025.1633961)

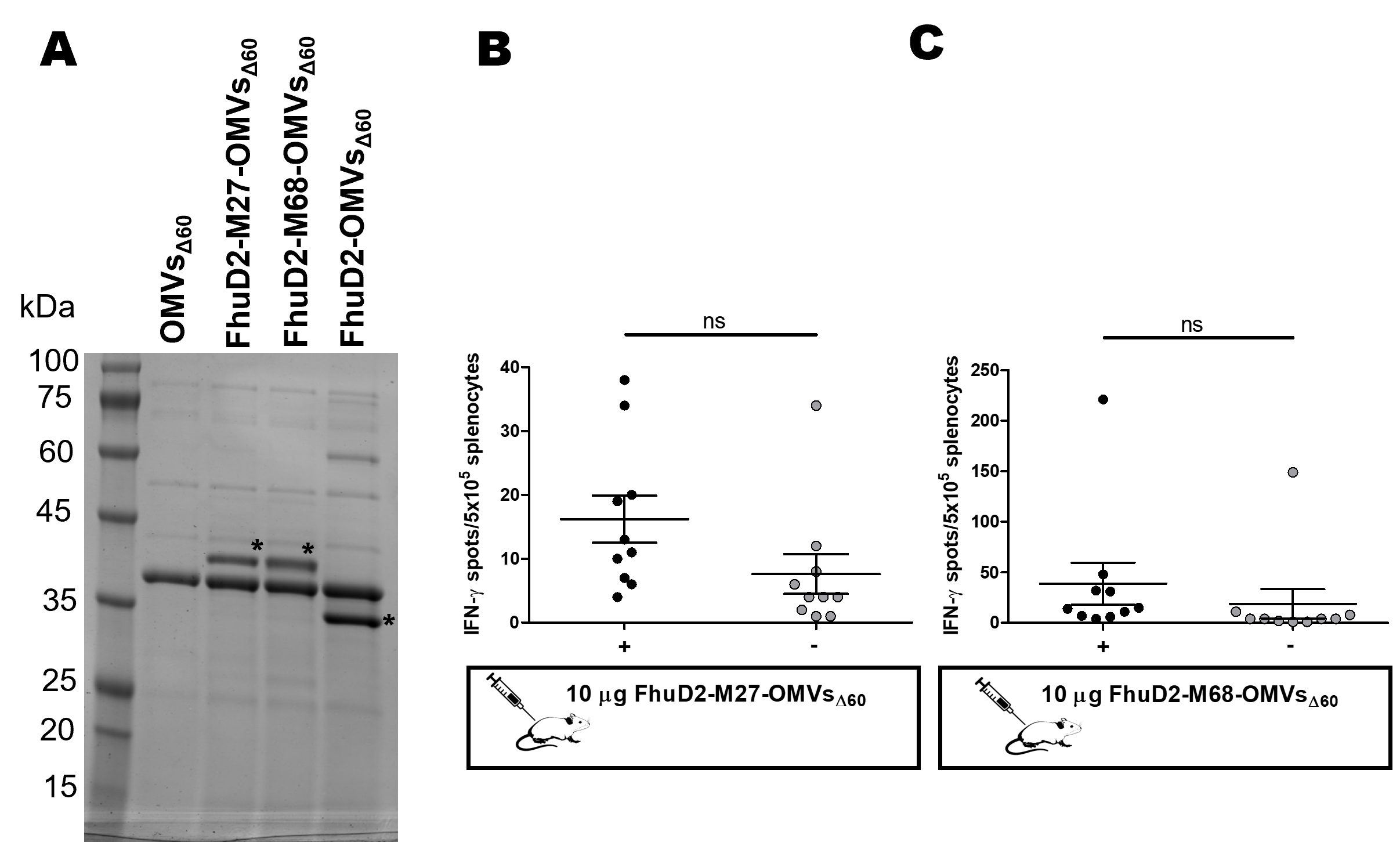

Supplement: Supplementary Figure 1 — Epitope-specific IFN-γ+ CD4+ T cells elicited by immunizations with OMVsΔ60 engineered with MHCII-restricted epitopes M27 and M68. (A) SDS-PAGE analysis of OMVsΔ60 engineered with M27 and M68 MHCII epitopes fused to the C-terminus of FhuD2 carrier protein (see Text for details). Purified OMVs (10 µg) were loaded on the gel and protein species were visualized by Coomassie Blue staining. The asterisks indicate the bands corresponding to the FhuD2-M27 fusion (lane 3), FhuD2-M68 fusion (lane 4) and to FhuD2 carrier protein (lane 5). Quantification of the fusion proteins expressed in OMVs was performed by densitometric analysis using Image Lab software (BioRad). (B, C) BALB/c C57BL/6 mice were i.p. immunized on day 0 and day 7 with 10 µg of OMVsΔ60 engineered with either FhuD2-M27-OMVsΔ60 (B) or 10 µg of FhuD2-M68-OMVsΔ60 (C) and after 5 days from the second immunization, splenocytes, were collected from each mouse and peptide‐specific IFN-γ+ CD4+ T cells were analyzed by ELISpot, after stimulation with the 5 µg/ml of peptide used in vaccination (black) or 5 µg/ml of an unrelated peptide (gray). Statistical significance was calculated using an unpaired, two-tailed Student’s t-test. ns, not significant, *P<0.1; **P<0.01; ***P<0.001. [file Image1.tif]
